# Are Cerebral White Matter Lesions Related to the Presence of Bilateral Internal Carotid Artery Stenosis or to the Length of Stenosis Among Patients With Ischemic Cerebrovascular Events?

**DOI:** 10.3389/fneur.2019.00919

**Published:** 2019-08-29

**Authors:** Ahmed Mohamed Elhfnawy, Jens Volkmann, Mira Schliesser, Felix Fluri

**Affiliations:** ^1^Department of Neurology, University Hospital Würzburg, Würzburg, Germany; ^2^Department of Neurology, University Hospital of Essen, Essen, Germany; ^3^Department of Neurology, University Hospital of Alexandria, Alexandria, Egypt; ^4^Department of Neurology, Kantonssptial St. Gallen, St. Gallen, Switzerland

**Keywords:** stroke, transient ischemic attack, white matter lesions, internal carotid artery stenosis, bilateral internal carotid artery stenosis, degree of stenosis, length of stenosis

## Abstract

**Background and purpose:** Previous studies delivered contradicting results regarding the relation between the presence of an internal carotid artery stenosis (ICAS) and the occurence of white matter lesions (WMLs). We hypothesize that special characteristics related to the ICAS might be related to the WMLs. We examined the relation between the presence of bilateral ICAS, the degree and length of stenosis and ipsi-, contralateral as well as mean white matter lesion load (MWMLL).

**Methods:** In a retrospective cohort, patients with ischemic stroke or transient ischemic attack (TIA) as well as ipsi- and/or contralateral ICAS were identified. The length and degree of ICAS, as well as plaque morphology (hypoechoic, mixed or echogenic), were assessed on ultrasound scans and, if available, the length was also measured on magnetic resonance angiography (MRA) scans, and/or digital subtraction angiography (DSA). The WMLs were assessed in 4 areas separately, (periventricular and deep WMLs on each hemispherer), using the Fazekas scale. The MWMLL was calculated as the mean of these four values.

**Results:** 136 patients with 177 ICAS were identified. A significant correlation between age and MWMLL was observed (Spearman correlation coefficient, *ρ* = 0.41, *p* < 0.001). Before adjusting for other risk factors, a significantly positive relation was found between the presence of bilateral ICAS and MWMLL (*p* = 0.039). The length but not the degree of ICAS showed a very slight trend toward association with ipsilateral WMLs and with MWMLL. In an age-adjusted multivariate logistic regression with MWMLL ≥2 as the outcome measure, atrial fibrillation (OR 3.54, 95% CI 1.12–11.18, *p* = 0.03), female sex (OR 3.11, 95% CI 1.19–8.11, *p* = 0.02) and diabetes mellitus (OR 2.76, 95% CI 1.16–6.53, *p* = 0.02) were significantly related to WMLs, whereas the presence of bilateral stenosis showed a trend toward significance (OR 2.25, 95% CI 0.93–5.45, *p* = 0.074). No relation was found between plaque morphology and MWMLL, periventricular, or deep WMLs.

**Conclusion:** We have shown a slight correlation between the length of stenosis and the presence of WMLs which might be due to microembolisation originating from the carotid plaque. However, the presence of bilateral ICAS seems also to be related to WMLs which may point to common underlying vascular risk factors contributing to the occurrence of WML.

## Introduction

White matter changes are commonly detected in the brains of elderly people (1–4) and are associated with cognitive changes, gait instability, and focal neurological signs, as well as bladder and bowel symptoms (4–6). Hypertension, diabetes mellitus, dyslipidemia, and smoking were found to be related to white matter lesions (WMLs) (5, 7, 8). Chronic hypertension is believed to induce lipohyalinosis of the small perforating cerebral arteries and arterioles and thus cause the development of WMLs (9). Several studies found an association between internal carotid artery stenosis (ICAS) and the ipsilateral WMLs, raising suspicion about an embolic origin, at least for some WMLs (1, 10, 11). Especially, unstable plaques were more related to the ipsilateral white matter lesions than stable plaques (11). Moreover, an index combining plaque echolucency, surface irregularity, and the degree of stenosis was found to better predict ischemic manifestations than the degree of stenosis alone (12). Another pathophysiological mechanism explaining this association came from animal models with induced ICAS, where chronic hemodynamic impairment caused the delayed development of WMLs (13, 14). On the other hand, several studies found no relation between ICAS, and WMLs, and hence it was suggested that both are the result of the underlying risk factors like age or hypertension (15, 16). To explain these contradicting results, we speculate that special characteristics related to the ICAS might be related to the WMLs and hence in absence of these characteristics, ICAS would not be associated with WMLs. Short segmental carotid stenosis induces more hemodynamic changes (17) resulting in the development of WMLs as shown in animal models. We hypothesize that long segmental carotid stenosis is associated with a higher plaque burden, which might be the source of microemboli which finally might lead to WMLs. In the current work, we aimed to study the relation between WMLs and the presence of bilateral carotid artery stenosis as well as the degree and length of stenosis. Bilateral ICAS may reflect the underlying risk factors, while more severe degree and increased length of stenosis may induce different patterns of hemodynamic changes or increase the risk of microembolism.

## Materials and Methods

### Inclusion and Exclusion Criteria

**Figure 1:**
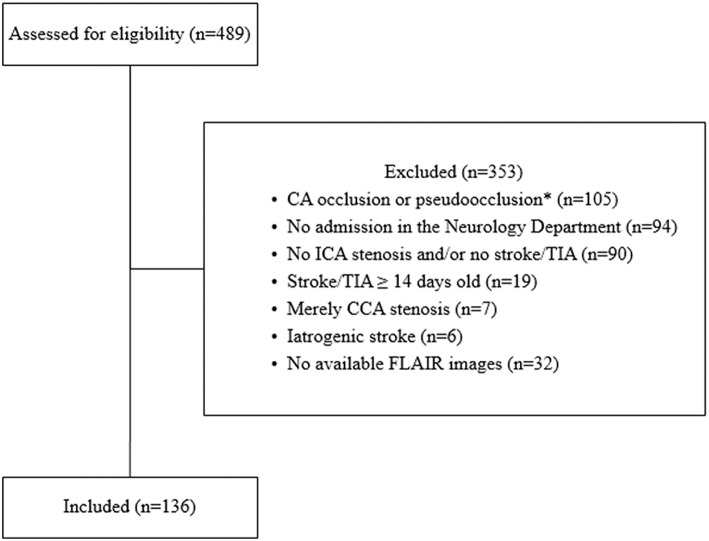
Flow chart showing the included and excluded patients in the current study. CA: carotid artery, CCA: common carotid artery, ICA: internal carotid artery, TIA: transient ischemic attack, iatrogenic stroke (5 cases after carotid endarterectomy, 1 case after coronary angiography). *Patients were excluded if no further ICA stenosis on the other side was detected. Of the 489 screened patients, 136 patients met our inclusion and exclusion criteria.

In this study, 136 patients admitted to the Department of Neurology, University Hospital of Würzburg in the period from January 2011 till September 2016 with either ischemic stroke or transient ischemic attack (TIA), in the presence of ipsilateral and/or contralateral cervical ICAS of ≥20% as detected by ultrasound methodology scans were retrospectively recruited. The exclusion criteria are shown in Figure 1.

### Assessment of the White Matter Lesions

Fazekas scale was used to assess the periventricular and the deep white matter lesion load (WMLL) on fluid-attenuated inversion recovery (FLAIR) sequence of magnetic resonance imaging (MRI) (2). According to Fazekas scale, periventricular white matter lesions can be classified into: 0 = absent, 1 = caps or pencil-thin lining, 2 = smooth “halo,” and 3 = irregular periventricular signal extending into the deep white matter, while deep white matter lesions are classified as: 0 = absent, 1 = punctate foci, 2 = beginning confluence, 3 = large confluent areas. The WMLs were assessed on every side separately as follows: 1. Periventricular (PV1) or deep white matter lesions (DWM1): ipsilateral to the symptomatic (sICAS) or, in absence of sICAS, the more stenotic side, 2. Periventricular (PV2) or deep white matter lesions (DWM2) contralateral to the sICAS or, in absence of sICAS, contralateral to the more stenotic side, 3. Mean white matter lesion load (MWMLL) defined as the mean of PV1, DWM1, PV2, and DWM2. The MWMLL was further stratified into <2 or ≥2. MRI scanners with a field strength of 1.5 or 3 Tesla were used according to the standardized stroke acquisition protocols in the University Hospital of Würzburg with a slice thickness of 5 mm and an interslice gap of 0.5 mm. Brain infarction was diagnosed in the strong (*b* = 1,000) diffusion-weighted images (DWI) of MRI.

### Measurement of the Degree and Length of Stenosis and Assessment of Plaque Morphology

**Figure 2:**
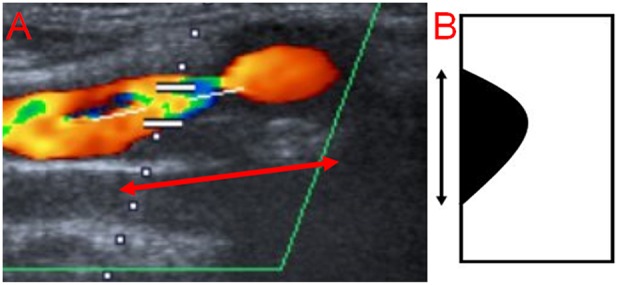
Measurement of the length of carotid stenosis from the most proximal to the most distal stenotic segment. **(A)** Ultrasound image (the length is represented by the red arrow with double head), **(B)** Schematic representation (the length is represented by the black arrow with double head).

The degree and length of stenosis, as well as plaque morphology, were assessed on ultrasound scans in our Picture Archiving and Communication System (PACS). The degree of ICAS was measured according to the hemodynamic criteria of the North American Symptomatic Carotid Endarterectomy Trial (NASCET) (17). The length was measured by a single non-blinded observer (AME) from the most proximal to the most distal stenotic segment in the available ultrasound images (Figure 2) and if available, on magnetic resonance angiography (MRA) scans, and/or digital subtraction angiography (DSA) in the projection showing the stenosis in its longest segment using a previously published method (18). Concerning sonography, the following criteria were used to identify the stenosis: 1. visible narrowing of the vascular lumen, 2. flow turbulence in the proximal stenotic end, provided that the ultrasound examiner used the appropriate color scheme (pulse repetition frequency). For the distal end, flow turbulence was used to identify the distal stenotic end, only in the presence of corresponding Doppler waves showing increased flow velocity. This is because the differentiation between flow turbulence due to increased flow velocity resulting from stenosis and post-stenotic flow turbulence is not possible in the absence of Doppler waves demonstrating the flow velocity, and 3. Severe calcification causing acoustic shadowing and interfering with visualization of the vascular wall. Regarding MRA and DSA, only the first criterion was used. Plaque echolucency was classified using a visual rating scale into hypoechoic, mixed or echogenic (19). The ultrasound examination was conducted with an AplioXG Toshiba ultrasound machine (Toshiba Medical Systems Corporation, Tochigi, Japan) using a 7.5 MHz linear array transducer.

### Ethics Statement

Data collected within routine clinical care were used. Therefore, no specific approval was needed according to local regulations confirmed by the Ethics Board of the Medical Faculty of the University of Würzburg. Our Ethical Committee was consulted before the conduction of the study and the need for informed consent was waived because of the retrospective nature of the study.

### Statistics

Quantitative data were expressed using median and interquartile range, while qualitative data were expressed in absolute and relative frequencies. To check for normality, we used graphical methods (QQ-plot and histogram) and the Shapiro-Wilk test. Univariable statistical tests were conducted using χ2 test for categorical data. Mann-Whitney *U*-test or Kruskal-Wallis Test were used for continuous data. Spearman coefficient was used to analyze correlations. A univariate binary regression analysis was performed and variables with *p* < 0.2 were further analyzed in a multivariate regression analysis model with inclusion method. Data were analyzed in SPSS software package version 25 (SPSS, Chicago IL USA). *P* < 0.05 were considered statistically significant.

## Results

**Figure 3:**
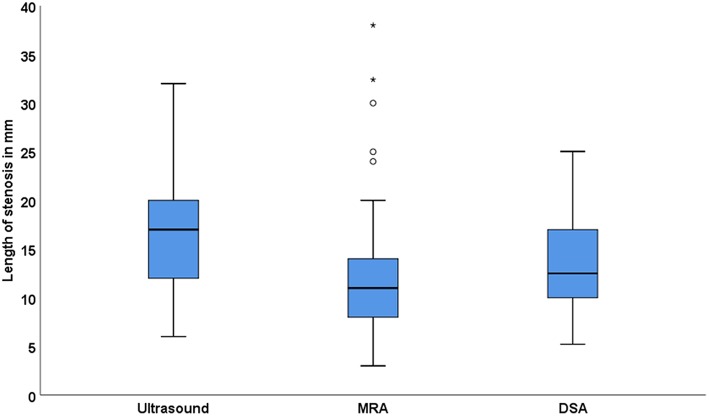
Length of internal carotid artery stenosis (mm) in ultrasound, MRA and DSA.

Four hundred and eighty nine patients were screened and 136 patients were included with 177 arteries with ICAS, i.e., 95 had unilateral and 41 bilateral ICAS. The baseline characteristics are shown in Table 1. We were able to scans measure the length of ICAS in 141 arteries on ultrasound scans, in 110 arteries on MRA scans and in 24 arteries on DSA images, whereas the degree was measured in 164 arteries on ultrasound scans. There was a significant correlation between the measurement of the length on ultrasound scans compared to that assessed on MRA scans (Spearman ρ = 0.33, *p* = 0.002, *n* = 89), on ultrasound vs. on DSA scans (ρ = 0.46, *p* = 0.07, *n* = 17) and on MRA vs. on DSA images (ρ = 0.47, *p* = 0.07, *n* = 16). Figure 3 shows the length of stenosis on ultrasound, MRA and DSA images.

**Table 1:** Baseline characteristics.

	**MWMLL <2 (*n* = 90)**	**MWMLL ≥ 2 (*n* = 46)**	***naOR (95% CI), p-value***	***aOR (95% CI), p-value***
Female sex, *n*%	20 (22.2)	19 (41.3%)	2.46 (1.14-5.31), *p* = 0.02^*^	3.11 (1.19-8.11), *p* = 0.02^*^
Age (y), median (IQR)	72 (65–76)	77 (69–82)	1.08 (1.032–1.13), 0.001^*^	1.08 (1.02–1.14), *p* = 0.006^*^
Hypertension, *n*%	77 (85.6)	43 (93.5)	2.42 (0.65–8.97), *p* = 0.19	1.54 (0.29–8.16), *p* = 0.61
Diabetes mellitus, *n*%	23 (25.6)	23 (50)	2.91 (1.38–6.15), *p* = 0.005^*^	2.76 (1.16–6.53), *p* = 0.02^*^
Atrial fibrillation, *n*%	7 (7.8)	13 (28.3)	4.67 (1.71–12.74), *p* = 0.003^*^	3.54 (1.12–11.18), *p* = 0.03^*^
Smoking status, *n*%			0.35 (0.13–0.91), *p* = 0.03^*^^†^	0.95 (0.29–3.09), *p* = 0.94^†^
Smoker	25 (27.8)	7 (15.2)		
Ex-smoker	26 (28.9)	9 (19.6)		
Non-smoker	37 (41.1)	30 (65.2)		
Na	2 (2.2)			
LDL-Cholesterol (mg/dl), median (IQR)	115.5 (87.8–141.3)	100 (80–136)	1 (0.99–1), *p* = 0.35	
HbA1c %, median (IQR)	5.9 (5.5–6.3)	6.3 (5.8–7.5)	1.62 (1.16–2.28), *p* = 0.005^*^	††
Bilateral ICAS, *n*%	23 (25.6)	18 (39.1)	1.87 (0.88–4), *p* = 0.11	2.25 (0.93–5.45), *p* = 0.074
Length of ICAS on the longer side (mm), median (IQR)				
Ultrasound	17 (13–20)	17 (13–22)	1.02 (0.95–1.09), *p* = 0.64	
MRA	11 (8–14)	12 (9–15)	1.02 (0.93–1.11), *p* = 0.7	
DSA	12 (8–14)	18 (14–21)	1.26 (0.99–1.6), *p* = 0.06	§
Degree of ICAS on the more stenotic side (%), median (IQR)	70 (20–80)	70 (20–90)	1.05 (0.87–1.25), *p* = 0.63	
Plaque morphology§§, *n*%				
Hypoechoic	36 (42.4)	18 (42.9)	1.02 (0.48–2.16), *p* = 0.96	
Mixed	37 (41.1)	19 (41.3)	0.79 (0.26–2.4), *p* = 0.68	
Echogenic	12 (13.3)	5 (10.9)	1.01 (0.49–2.07), *p* = 0.98	
Stroke rather than TIA§§§, *n*%	65 (72.2)	35 (76.1)	1.22 (0.54–2.78), *p* = 0.63	
NIHSS^§§§^, median (IQR)	1 (0–3)	3 (0–5)		
NIHSS ≥ 4, *n*%	19 (21.1)	22 (47.8)	3.43 (1.59–7.39), 0.002^*^	
NIHSS ≥ 8, *n*%	4 (4.4)	8 (17.4)	4.53 (1.29–15.95), *p* = 0.02^*^	

*Results are expressed as frequencies (%) or median (interquartile range); aOR, adjusted odds ratio using a multivariate regression model including all factors under this column; DSA, digital subtraction angiography; HbA1c, Hemoglobin A1c; ICAS, internal carotid artery stenosis; IQR, interquartile range; LDL-cholesterol, low density lipoprotein cholesterol; MRA, magnetic resonance angiogram; MWMLL, mean white matter lesion load; na, not available; naOR, non-adjusted odds ratio; TIA, transient ischemic attack*;

* Statistically significant results;

† Odds ratio calculated for smokers versus non-smokers; N.b, the non-adjusted OR was statistically significant, after adjusting for age and sex, OR was not significant because of the strong positive association between age and MWMLL and smokers were younger in age;

†† DM was already included in the model;

§ This variable was not included in the model because we had only 21 cases with available DSA;

§§ Plaque of the internal carotid artery stenosis on the symptomatic side or, in absence of symptomatic stenosis, the more stenotic side, in 9 arteries the assessment of plaque morphology was not possible;

§§§ *In these models, the relevant factors were outcomes and the white matter lesions was the predictor*.

### Correlations of WMLs With the Length and Degree of ICAS as Well as Plaque Morphology

We did not find any statistically significant correlation between the length of the ICAS, measured with any of these three examination modalities (ultrasound, MRA and DSA) and the white matter lesions. Moreover, we found no correlation between the degree of ICAS and the WMLL. The various correlations performed are shown in Tables 2, 3.

**Table 2:** Correlation between the length of ICAS and white matter lesion load (WMLL).

**Method**	**L1 with PV1**	**L1-S with PV1**	**L1 with DWM1**	**L1-S with DWM1**	**L2 with PV2**	**L2 with DWM2**	**L1 with PV2**	**L1 with DWM2**	**MWMLL with longer side**
Duplex	ρ = 0.08^*^	ρ = 0.14	ρ = 0.07	ρ = 0.16	ρ = 0.03	ρ = 0.01	ρ = 0.03	ρ = −0.004	ρ = 0.07
	(*p =* 0.40)	(*p =* 0.19)^†^	(*p =* 0.45)	(*p =* 0.14)^†^	(*p =* 0.90)	(*p =* 0.96)	(*p =* 0.77)	(*p =* 0.97)	(*p =* 0.42)
	*N =* 116	*N =* 88	*N =* 116	*N =* 88	*N =* 25	*N =* 25	*N =* 116	*N =* 116	*N =* 122
MRA	*ρ =* 0.13	*ρ =* 0.16	*ρ =* 0.18	*ρ =* 0.20	*ρ =* −0.34	*ρ =* −0.07	*ρ =* 0.11	*ρ =* 0.12	*ρ =* 0.19
	(*p =* 0.24)	(*p =* 0.20)^†^	(*p =* 0.11)^†^	(*p =* 0.11)^†^	(*p =* 0.08)^†^	(*p =* 0.73)	(*p =* 0.31)	(*p =* 0.31)	(*p =* 0.09)^†^
	*N =* 82	*N =* 71	*N =* 82	*N =* 71	*N =* 28	*N =* 28	*N =* 82	*N =* 82	*N =* 83
DSA	*ρ =* 0.38	*ρ =* 0.35	*ρ =* 0.21	*ρ =* 0.15	*ρ =* 0.5	*ρ =* 0.87	*ρ =* 0.25	*ρ =* 0.30	*ρ =* 0.31
	(*p =* 0.09)^†^	(*p =* 0.16)^†^	(*p =* 0.36)	(*p =* 0.54)	(*p =* 0.67)	(*p =* 0.33)	(*p =* 0.28)	(*p =* 0.19)^†^	(*p =* 0.18)^†^
	*N =* 21	*N =* 18	*N =* 21	*N =* 18	*N =* 3	*N =* 3	*N =* 21	*N =* 21	*N =* 21

* *Spearman correlation coefficient (ρ)*,

† *very slight trend toward significance, L1, the length of internal carotid artery stenosis on the symptomatic side or; in absence of symptomatic stenosis; the more stenotic side; L1-S, the length of internal carotid artery stenosis on the symptomatic side; L2, the length of internal carotid artery stenosis on the asymptomatic side in patients with bilateral carotid stenosis; PV1 and DWM1, periventricular and deep white matter lesions; respectively ipsilateral to L1; PV2 and DWM2, periventricular and deep white matter lesions respectively contralateral to L1; MWMLL, mean white matter lesion load; DSA, digital subtraction angiography MRA, magnetic resonance angiogram*.

**Table 3:** Correlation between the degree of ICAS and white matter lesion load (WMLL).

**Method**	**D1 with PV1**	**D1-S with PV1**	**D1 with DWM1**	**D1-S with DWM1**	**D2 with PV2**	**D2 with DWM2**	**D1 with PV2**	**D1 with DWM2**	**MWMLL with more stenotic side**
Duplex	*ρ =* 0.003^*^	*ρ =* 0.09	*ρ =* 0.04	*ρ =* 0.07	*ρ =* −0.19	*ρ =* −0.02	*ρ =* 0.05	*ρ =* 0.08	*ρ =* 0.05
	(*p =* 0.98)	(*p =* 0.38)	(*p =* 0.64)	(*p =* 0.47)	(*p =* 0.31)	(*p =* 0.92)	(*p =* 0.56)	(*p =* 0.34)	(*p =* 0.55)
	*N =* 134	*N =* 103	*N =* 134	*N =* 103	*N =* 30	*N =* 30	*N =* 134	*N =* 134	*N =* 134

* *Spearman correlation coefficient (ρ), D1, the degree of internal carotid artery stenosis on the symptomatic side or, in absence of symptomatic stenosis, the more stenotic side, D1-S, the degree of internal carotid artery stenosis on the symptomatic side; D2, the degree of internal carotid artery stenosis on the asymptomatic side in patients with bilateral carotid stenosis; PV1 and DWM1, periventricular and deep white matter lesions respectively ipsilateral to D1; PV2 and DWM2, periventricular and deep white matter lesions; respectively contralateral to D1; MWMLL, mean white matter lesion load*.

We found a very slight trend toward a positive relation between the length of internal carotid artery stenosis on the symptomatic side (L1-S) and PV1, when the length was measured on duplex, MRA or DSA scans. The same was true for DWM1 when the length was measured on duplex or MRA scans. Furthermore, the length of the longer stenotic side, measured on MRA or DSA scans showed another slight trend toward a significant relation with the MWMLL. On the other hand, when performing these correlations with the degree of ICAS, none of the above-mentioned trends were observed.

Before adjusting for other risk factors, a statistically significant relation was found between the presence of bilateral ICAS and the MWMLL (*p* = 0.039). In an age-adjusted multivariate logistic regression model with MWMLL ≥ 2 as the outcome measure, atrial fibrillation (OR 3.54, 95% CI 1.12–11.18, *p* = 0.03), female sex (OR 3.11, 95% CI 1.19–8.11, *p* = 0.02), and diabetes mellitus (OR 2.76, 95% CI 1.16–6.53, *p* = 0.02) and were significantly related to the WMLs, whereas the presence of bilateral stenosis showed a considerable trend toward significance (OR 2.25, 95% CI 0.93–5.45, *p* = 0.074). Hypertension (OR 1.54, 95% CI 0.29–8.16, *p* = 0.61) and smoking (OR 0.95, 95% CI 0.29–3.09, *p* = 0.94) were not related to WMLs (Table 1).

**Figure 4:**
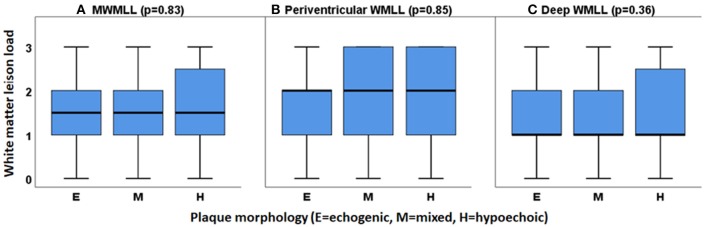
Relation between the three types of plaque morphology (hypoechoic, mixed and echogenic) with white matter lesion load (WMLL). **(A)** MWMLL, Mean white matter lesion load (*p* = 0.83), **(B)** Periventricular white matter lesion load (*p* = 0.85), **(C)** Deep white matter lesion load (*p* = 0.36).

No association was observed between plaque morphology and WMLs. Especially, no correlation was found when testing the association between white matter lesion load; this was true for MWMLL, periventricular and deep WMLs, and any single plaque morphology in comparison to the other two plaque morphologies (e.g., hypoechoic vs. non-hypoechoic) using the Mann-Whitney *U*-test or when investigating the relation between WMLs and the three plaque morphologies, i.e., hypoechoic vs. mixed vs. echogenic using the Kruskal-Wallis test. The relations between the three plaque morphologies and the white matter lesion load are shown in Figure 4.

### Associations Between MWMLL and Other Factors

We found a statistically significant relationship between MWMLL and the following parameters: age (Spearman correlation, ρ = 0.41, *p* < 0.001), severity of stroke assessed by NIHSS on admission (ρ = 0.23, *p* = 0.02), duration of hospital stay (ρ = 0.19, *p* = 0.03), and serum levels of HbA1c (ρ = 0.26, *p* = 0.003). Moreover, a statistically significant correlation was found between MWMLL and diabetes mellitus (median MWMLL for diabetics 2 vs. 1.25 for non-diabetics, *p* = 0.006), atrial fibrillation (AF) (median MWMLL for patients without AF 1.5 vs. 2 for patients with AF, *p* = 0.03), hypertension (median MWMLL for non-hypertensive patients 1.25 vs. 1.5 for hypertensive patients, *p* = 0.048) as well as female sex (median MWMLL for males 1.25 vs. 1.75 for females, *p* = 0.049). On the other side, a statistically significant inverse relation was observed between smoking status and MWMLL (median for non-smokers 1.75, for ex-smokers 1.50 and for smokers 1, *p* = 0.04). This might be explained by the strong positive association between age and MWMLL and the younger age of smokers (median age for non-smokers 77 years, for ex-smokers 73 years and for smokers 63 years, *p* < 0.001), and hence this relation was not observed in our age-adjusted multivariate regression model (OR 0.95, 95% CI 0.29–3.09, *p* = 0.94). No correlation was found between the MWMLL and LDL-Cholesterol (ρ = 0.03, *p* = 0.76).

## Discussion

### Correlation Between ICAS and WMLs

We found a statistically significant relation between the presence of bilateral ICAS and the WMLL before adjusting for other risk factors. After adjusting for other risk factors, this relation showed a trend toward significance. No statistically significant correlation was found between the length or the degree of ICAS and the ipsi- or contralateral WMLL, neither for periventricular nor for deep WMLs. However, it seems that there is a very slight but not significant correlation between periventricular and deep WMLL, as well as symptomatic stenoses, and the length of ICAS. Increasing the study population in future studies might show a significant relationship in statistical terms. Our findings are in line with those of Kwee et al. (10), reporting a significant correlation between the total carotid plaque volume and ipsilateral WMLs (ρ = 0.39, *P* = 0.005). Plaque length and degree of stenosis are components of the carotid plaque volume. However, it was previously shown that an inverse relation exists between the plaque length and degree of stenosis among patients with ICAS ≥70% (18). In other words, totally different plaque lengths may result in the same plaque volume. Therefore, it is important to study each component separately. In a large population-based study, the periventricular but not deep WMLs were significantly associated with an increased number of plaques in the carotid artery (20). On the other hand, several studies found no relation between carotid atherosclerosis and white matter lesions (15, 16). For example, Potter et al. found no association between ipsi- or contralateral ICAS and WMLs, before or after adjusting for other risk factors and suggested that the association between ICAS and WMLs are due to these underlying risk factors (15). In our study, WMLs were more prominent in patients with bilateral ICAS. This might support the theory of comparable underlying risk factors. Similar to previous studies (1, 10), we found no relation between the degree of ICAS and the WMLs.

In the current work, no relation was found between plaque morphology and WMLs. Specifically, the high-risk echolucent plaques were not related to the WMLs in our cohort. Of note, echolucent plaques are characterized by intraplaque hemorrhage (21). Similarly, previous studies found no association between ipsilateral white matter lesions and plaques with a high vulnerability like those with lipid-rich necrotic core, intraplaque hemorrhage, or thin and/or ruptured fibrous cap (10). In contrast, previous studies found that unstable type VI plaques, defined according to the American Heart Association (AHA) histological classification, are more likely associated with at least double ipsilateral WMLL than stable type V plaques (11). Similar to echolucent plaques, type VI plaque is characterized by intraplaque hemorrhage (11). The aforementioned study suggests that some WMLs might have a microembolic arterial origin. We propose that future studies should directly investigate the relation between microembolic signals on transcranial ultrasound and WMLs.

Short segmental carotid stenosis induces more hemodynamic disturbances (17). In experimental animal models, chronic hemodynamic compromise caused delayed development of WMLs (13, 14). We speculate that the development of WMLs in patients with short segmental ICAS might be hemodynamically related. We further assume that long segmental ICAS might be associated with more plaque burden, and hence induce white matter lesions due to microembolisation. The aforementioned studies pointing to the relation between total carotid plaque volume, the number of plaques and unstable plaques with white matter lesions might support this hypothesis (10, 11, 20).

It might be postulated that specific characteristics of ICAS like the presence of bilateral ICAS or the length of ICAS might be related to WMLs. Furthermore, the origin of WMLs may be multifactorial. This might explain the contradictory results of the previous studies.

### Ultrasound Length in Different Examination Modalities

We found that ICAS is longer in ultrasound, followed by DSA and shortest in MRA. A significant correlation was found between the length of ICAS measured on ultrasound scans compared to that assessed on MRA images probably due to the small sample size, a trend for a positive correlation was observed between measurements of ICAS-length on ultrasound scans and that performed on DSA images (*p* = 0.07, *n* = 17) as well as between assessments of ICAS-length on MRA images and that on DSA images (*p* = 0.07, *n* = 16). We believe that this difference is modality-specific, i.e., the plaque can be better visualized on ultrasound scans than using any other imaging modality (18). On the other side, there is a tendency for MRA to overestimate the degree of stenosis (18).

### Atrial Fibrillation and Carotid Artery Stenosis; Two Different Diseases but Similar Effects

In line with previous studies (22, 23), we found that AF is significantly related to WMLs. It has been previously suggested that the relation between AF and WMLs may be related to: 1. similar underlying risk factors, 2. cerebral hypoperfusion resulting from decreased cardiac output, or 3. cardioembolism (22, 24). These 3 pathophysiological mechanisms may be more or less similar to those explaining the relation between ICAS and WMLs. Of note, the stroke risk is increased by 5-fold in patients with AF (25). The risk of AF-related stroke increases significantly with age; the risk is around 1.5% at the age of 50–59 years and is as high as 25.5% at 80–89 years (25).

### Study Limitations

We are aware of the following limitations. First, the lack of a control group without carotid plaques or stenosis attenuates the significance of this study. It would be of interest to compare patients having unilateral ICAS with a control group of patients without ICAS. Second, the retrospective nature of this single-center study represents another shortcoming. Third, the small sample size is another limitation. However, our current sample size serves as a proof-of-concept. The replication of this work in an epidemiologic multi-center study might help to clarify this issue.

## Conclusions

WMLs might be related to specific characteristics of the ICAS rather than to the presence or absence of ICAS, which might explain the contradictory results of the current literature. Especially, the presence of bilateral internal carotid artery stenosis seems to be related to the white matter lesions. Furthermore, it appears that a weak relation exists between the length but not the degree of ICAS and the ipsilateral white matter lesions, which did not reach a statistical significance in this cohort. Moreover, the origin of WMLs may be multifactorial. We suggest that future studies should focus on examining the relation between specific characteristics of ICAS rather than ICAS per se and WMLs.

## Data Availability

The raw data supporting the conclusions of this manuscript will be made available by the authors, without undue reservation, to any qualified researcher.

## Ethics Statement

Data collected within routine clinical care were used. Therefore, no specific approval was needed according to local regulations confirmed by the Ethics Board of the Medical Faculty of the University of Würzburg. Our Ethical Committee was consulted before the conduction of the study and the need for informed consent was waived because of the retrospective nature of the study.

## Author Contributions

All authors made a substantial contribution to the conception, design, and revision of the draft. AE collected the data, performed the measurements and the statistical analysis and wrote the first draft. MS was the main ultrasound examiner in the study period. JV, MS, and FF supervised the work, provided consultations and revised the manuscript. All authors were involved in the final approval of the version to be published.

### Conflict of Interest Statement

The authors declare that the research was conducted in the absence of any commercial or financial relationships that could be construed as a potential conflict of interest.
