# Corrigendum: Are Cerebral White Matter Lesions Related to the Presence of Bilateral Internal Carotid Artery Stenosis or to the Length of Stenosis Among Patients With Ischemic Cerebrovascular Events?

**DOI:** 10.3389/fneur.2019.01058

**Published:** 2019-10-04

**Authors:** Ahmed Mohamed Elhfnawy, Jens Volkmann, Mira Schliesser, Felix Fluri

**Affiliations:** ^1^Department of Neurology, University Hospital Würzburg, Würzburg, Germany; ^2^Department of Neurology, University Hospital of Essen, Essen, Germany; ^3^Department of Neurology, University Hospital of Alexandria, Alexandria, Egypt; ^4^Department of Neurology, Kantonssptial St. Gallen, St. Gallen, Switzerland

**Keywords:** stroke, transient ischemic attack, white matter lesions, internal carotid artery stenosis, bilateral internal carotid artery stenosis, degree of stenosis, length of stenosis

In the original article, we neglected to include the funder “German Research Foundation (DFG)” and the “University of Wuerzburg (funding program Open Access Publishing)”. A **Funding** statement has therefore been added to the article:

“This study was funded by the German Research Foundation (DFG) and the University of Wuerzburg (funding program Open Access Publishing).”

The authors apologize for this error and state that this does not change the scientific conclusions of the article in any way. The original article has been updated.

